# Supplementary material for: Insecticide resistance and population structure of the invasive malaria vector, Anopheles stephensi, from Fiq, Ethiopia
Source: Sci Rep. 2024 Nov 11;14:27516. doi: 10.1038/s41598-024-78072-4 (PMC11554808; doi:10.1038/s41598-024-78072-4)
Supplement: Supplementary file 1 — Supplementary Information. [file 41598_2024_78072_MOESM1_ESM.pdf]

Insecticide resistance and population structure of the invasive malaria vector, *Anopheles stephensi*, from Fiq, Ethiopia

Jeanne N. Samake[1,2*], Solomon Yared[3*], Mussie Abdosh Hassen[4], Sarah Zohdy[2,5], Tamar E. Carter[1**]

1. Department of Biology, Baylor University, Waco, TX, USA
2. Entomology Branch, U.S. Centers for Disease Control and Prevention, Atlanta, GA, USA
3. Department of Biology, Jigjiga University, Jigjiga, Ethiopia
4. Somali Region Health Bureau, Jigjiga, Ethiopia
5. U.S. President's Malaria Initiative, Entomology Branch, U.S. Centers for Disease Control and Prevention, Atlanta, GA, USA

*These authors contributed equally

**Corresponding author

Correspondence to tamar_carter@baylor.edu.

Supplementary information

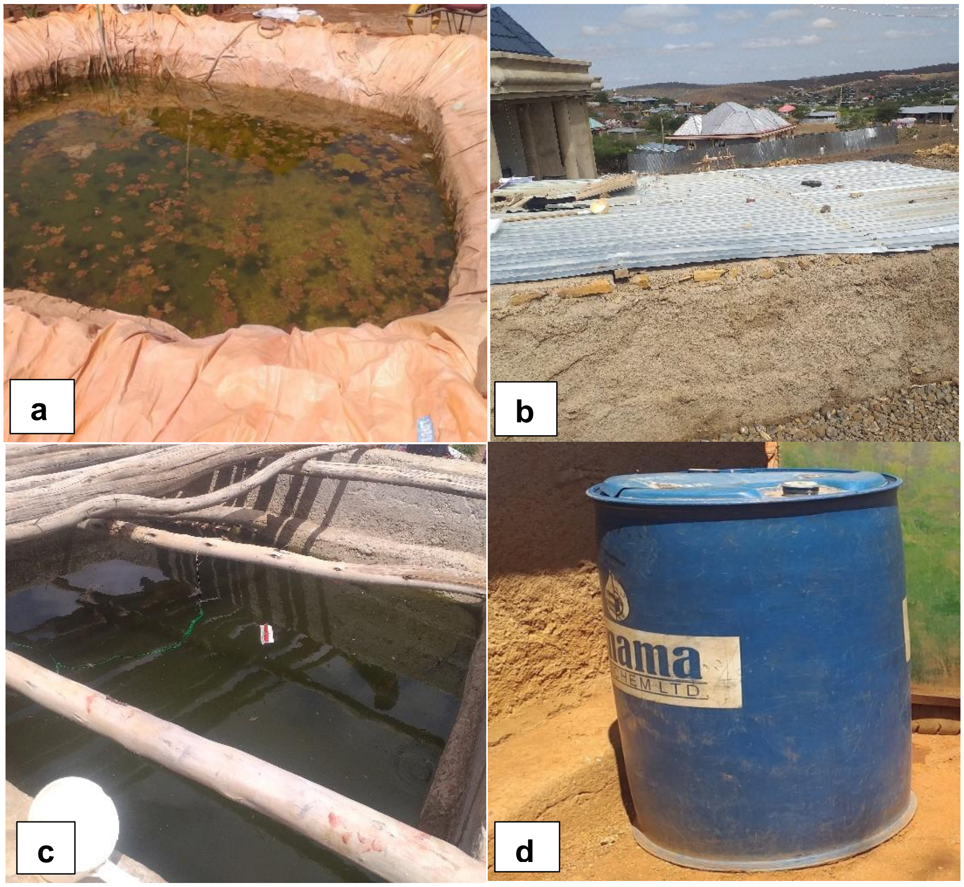

Figure S1. Breeding habitat types: **a.** Plastic sheet water storage **b.** Covered cistern **c.** Uncovered cistern **d.** Barrel

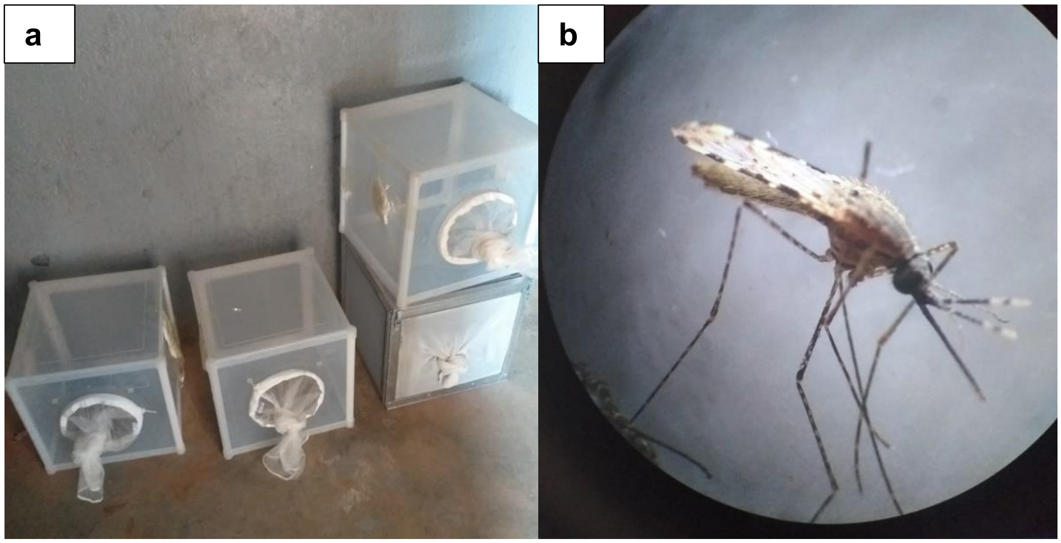

Figure S2. a. Rearing of *An. stephensi* at field insectary. **b.** Adult *An. stephensi* reared from wild larvae and pupae.

Table S1. Adult mosquito collection

**Collection methods**	**Fiq**	
	An. stephensi	*Culex* spp.
CDC LT	0	123
PSC	0	87
Prokopack (Goat shed)	2	9
Total	2	219

Table S2. Types of mosquito larval breeding habitats in Fiq town

Type of container	Number of containers inspected	Number of positive containers	Total larvae		Number of adult mosquitoes reared from *Anopheles* larvae
			Anopheles	*Culex*	*An. stephensi*
Cisterns	17	15	2066	132	2066
Plastic containers	8	8	1423	181	1423
Barrels	6	3	224	77	224
Total	31	26	3713	390	3713

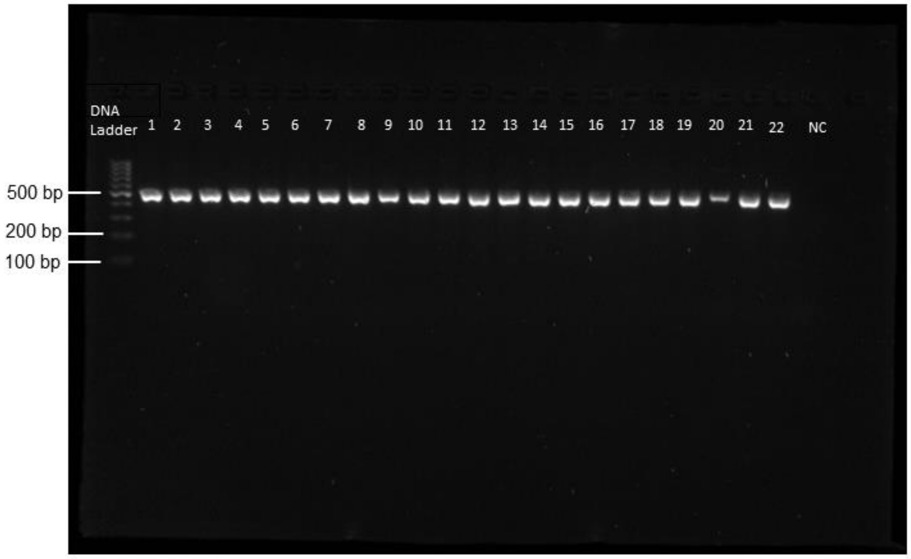

Figure S3. Gel photograph showing the result of ITS2 endpoint assay. Lanes 1-22 represent DNA samples from morphologically identified *An. stephensi* from Fiq, Ethiopia. NC stands for negative control.

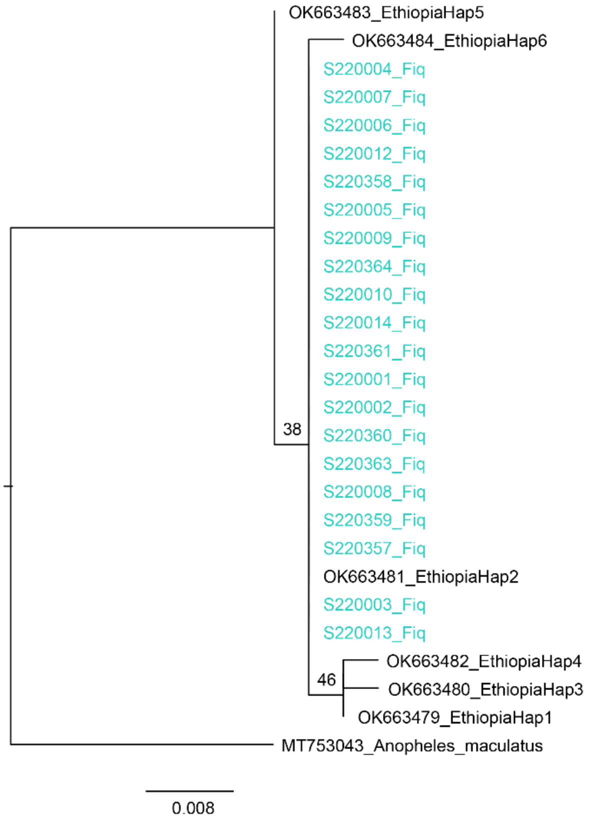

Figure S4. Phylogenetic analysis of Ethiopian *An. stephensi* cytochrome oxidase subunit I (COI) sequenced data based on maximum likelihood approach. The tree with the highest likelihood score is shown. Fiq *An. stephensi* sequences are shown in color.

Table S3. Mortality rates of *An. stephensi* exposed to different insecticides in Fiq, Ethiopia

**Type of assay**	**Classification**	**Insecticides discriminating concentration (%)**	**No. of *An. stephensi* tested**	**Mortality rate after 24hrs (%)**				**Mortality rate (%) mean [95% CI]**	**Resistance (<90%)**
				**R1**	**R2**	**R3**	**R4**		
Diagnostic dose	Carbamates	Bendiocarb 0.1%	100	40	40	32	48	40 [30, 50]	Yes
	Pyrethroid	Cyfluthrin 0.15%	100	96	76	88	88	87 [74, 100]	Yes
		Deltamethrin 0.05%	100	56	64	64	80	66 [50, 82]	Yes
		Permethrin 0.75%	100	52	60	64	76	63 [47, 79]	Yes
		Alphacypermethrin 0.05%	100	76	52	40	16	46 [6, 86]	Yes
	Organophosphorus	Pirimiphos-methyl 0.25%	100	100	100	100	100	100	No
Synergist assays	Pyrethroid	Deltamethrin 0.05%	75	88	28	48	-	55 [-21, 100]	Yes
		Deltamethrin 0.05% + PBO	75	100	100	100	-	100	No
		Permethrin 0.75%	75	84	80	48	-	71 [22, 100]	Yes
		Permethrin 0.75% + PBO	75	100	100	96	-	99 [93, 100]	No

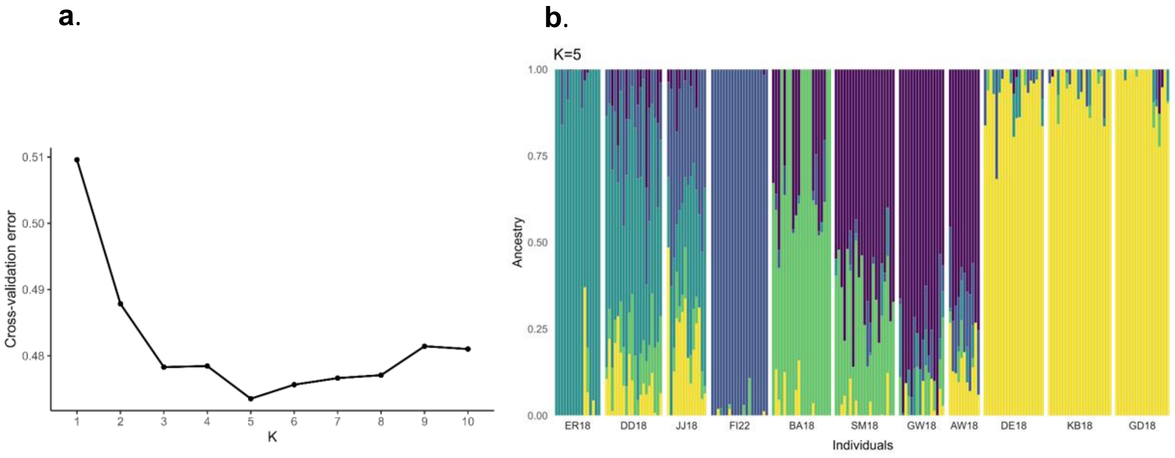

Figure S5. a. Admixture cross-validation errors of K values. **b.** Ancestry plot based on ADMIXTURE analysis. Each barplot represents an individual. The height of each colored bar represents the probability of assignment to the corresponding ancestry when assuming the presence of five ancestral populations (K = 5). The sample sites are Erer Gota (ER18), Dire Dawa (DD18), Jigjiga (JJ18), Fiq (FI22), Bati (BA18), Semera (SM18), Gewane (GW18), Awash Sebat Kilo (AW18), Degehabur (DE18), Kebriderar (KB18), and Godey, (GD18).

		Northeastern Ethiopia				East-central Ethiopia			Southeastern Ethiopia			Fiq
		Semera	Bati	Gewane	Awash Sebat Kilo	Erer Gota	Dire Dawa	Jigjiga	Degehabur	Kebridehar	Godey	Fiq
Northeastern Ethiopia	Semera	.	0.05	0.05	0.07	0.10	0.06	0.08	0.12	0.11	0.10	0.12
	Bati	0.05	.	0.08	0.10	0.13	0.08	0.10	0.14	0.14	0.14	0.14
	Gewane	0.05	0.08	.	0.06	0.09	0.06	0.08	0.11	0.11	0.10	0.12
	Awash Sebat Kilo	0.07	0.10	0.06	.	0.10	0.07	0.08	0.10	0.10	0.10	0.10
East-central Ethiopia	Erer Gota	0.10	0.13	0.09	0.10	.	0.06	0.08	0.12	0.12	0.12	0.12
	Dire Dawa	0.06	0.08	0.06	0.07	0.06	.	0.04	0.09	0.08	0.08	0.08
	Jigjiga	0.08	0.10	0.08	0.08	0.08	0.04	.	0.08	0.08	0.08	0.07
Southeastern Ethiopia	Degehabur	0.12	0.14	0.11	0.10	0.12	0.09	0.08	.	0.04	0.05	0.11
	Kebridehar	0.11	0.14	0.11	0.10	0.12	0.08	0.08	0.04	.	0.04	0.11
	Godey	0.10	0.14	0.10	0.10	0.12	0.08	0.08	0.05	0.04	.	0.13
Fiq	Fiq	0.12	0.14	0.12	0.10	0.12	0.08	0.07	0.11	0.11	0.13	.

Figure S6. Population pairwise F_{st} based on autosomal loci. Color gradient based on F_{st} values (yellow = highest, dark orange = lowest).

Table S4. Basic summary statistics based on autosomal loci.

Collection site	Sample size N	Nucleotide diversity π	Counts of segregating SNPs S	Tajima's D
**Fiq Ethiopia**				
Fiq	20	0.1986	1071	1.29
**Northeastern Ethiopia**				
Semera	21	0.2255	1374	0.76
Bati	21	0.2084	1218	0.95
Gewane	16	0.2225	1291	0.71
Awash Sebat Kilo	11	0.2300	1211	0.74
**East-central Ethiopia**				
Erer Gota	16	0.2195	1197	0.97
Dire Dawa	20	0.2261	1366	0.70
Jigjiga	14	0.2107	1198	0.66
**Southeastern Ethiopia**				
Degehabur	21	0.2018	1084	1.36
Kebridehar	22	0.2087	1101	1.48
Godey	18	0.1979	1078	1.19

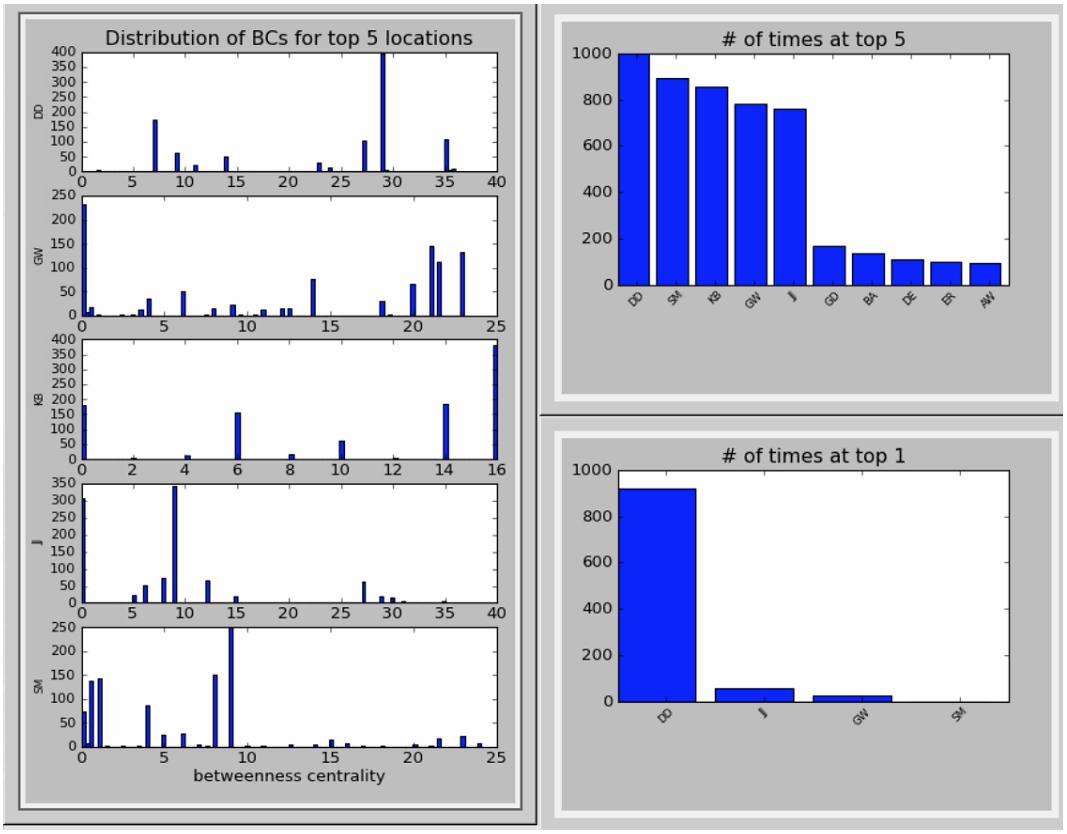

Figure S7. Bootstrapping results for betweenness centrality with number of repetitions (1000) and thresholded at 0.85 for Fiq, Ethiopia and eastern Ethiopia *An. stephensi* genetic networks.

Table S5. Fiq *An. stephensi kdr* mutation status.

Fiq samples	Insecticide	Status	KDR mutation/variant
1. S220007	Deltamethrin	Alive	No
2. S220008	Deltamethrin	Alive	No
3. S220009	Deltamethrin	Dead	No
4. S220010	Deltamethrin	Dead	No
5. S220012	Permethrin	Alive	No
6. S220013	Permethrin	Dead	No
7. S220014	Permethrin	Dead	No
8. S220357	Alphacypermethrin	Dead	No
9. S220358	Alphacypermethrin	Dead	No
10. S220359	Alphacypermethrin	Alive	Yes/heterozygous L1014F
11. S220360	Alphacypermethrin	Alive	No
12. S220361	Cyfluthrin	Dead	No
13. S220363	Bendiocarb	Dead	No
14. S220364	Bendiocarb	Alive	No
15. S220005	Pirimiphos-methyl	Dead	No
16. S220006	Pirimiphos-methyl	Dead	No
17. S220001	PBO+Deltamethrin	Dead	No
18. S220002	PBO+Deltamethrin	Dead	No
19. S220003	PBO+Permethrin	Dead	No
20. S220004	PBO+Permethrin	Dead	No

Supplementary Document S1

ddRAD-seq Library Preparation Methods

Genome-wide single nucleotide polymorphism (SNP) data was collected using the double-digest restriction-site associated DNA sequencing (ddRADseq) protocol outlined in Lavretsky et al. (2015), but with fragment size selection following Hernandez et al. (2021). A total of 20 samples with ~20 ng of genomic DNA each were enzymatically fragmented using 1 µL each of SbfI and EcoRI restriction enzymes. Illumina TruSeq compatible adapters and 6 base-pair barcodes were ligated to allow for future de-multiplexing. The adapter-ligated DNA fragments were then size selected using a double-sided size selection based on a total of 0.8x solution of sparQ PureMag beads (Quantabio, MA, USA). First, a right-sided selection for large fragments was completed by adding 0.55x concentration of the total starting ligated DNA solution. The solution was incubated at room temperature for 10 minutes, then transferred to a magnetic plate to rest for 5 minutes or until the mixture became clear. The supernatant containing both target-sized and small-sided DNA fragments was then transferred to new tubes and the beads were discarded. Next, a left-sided size selection against small DNA fragments (<100bp) was completed by adding 0.25x concentration of the total starting ligated DNA solution. The solution was again incubated at room temperature for 10 minutes, then transferred to a magnetic plate to rest for 5 minutes at room temperature or until the mixture became clear. The supernatant containing small-sided DNA fragments (<100bp) was then discarded and the beads were washed twice with 70% ethanol. The beads were then air-dried at room temperature for 2-5 minutes. DNA was re-suspended with 22 µL molecular grade water and eluted for 1 hour, then transferred to a magnetic plate to rest for 1 minute at room temperature or until the mixture became clear. The eluant was then transferred to new tubes. Target size selected DNA fragments were then PCR amplified with Phusion High-Fidelity DNA polymerase, and 10x concentration of forward and reverse RAD primers (Dacosta & Sorenson, 2014) under the following PCR conditions: an initial 30 sec cycle at 98 ^{o}C, followed by 22 cycles of 10 sec at 98 ^{o}C, 30 sec at 60 ^{o}C, and 40 sec at 72 ^{o}C, with a final extension at 72 ^{o}C for 5 min. Amplicons were then cleaned using a 1.8x solution of sparQ PureMag beads (Quantabio, MA, USA) and two 70% ethanol washed before final elution in 40 µL molecular grade water. Library concentrations were quantified using Qubit

dsDNA BR Assay Kit (ThermoFisher Scientific, MA, USA) following manufacturer protocols. Samples were then pooled in equimolar amounts and the multiplexed library was sequenced on Illumina Hiseq X using single-end 150 bp chemistry with Novogene (Novogene Inc., CA, USA).

REFERENCES

DaCosta, J. M., & Sorenson, M. D. (2014). Amplification biases and consistent recovery of loci in a double-digest RAD-seq protocol. PLoS ONE, 9(9), e106713. doi:10.1371/journal.pone.0106713

Hernández, F., Brown, J. I., Kaminski, M., Harvey, M. G., & Lavretsky, P. (2021). Genomic Evidence for Rare Hybridization and Large Demographic Changes in the Evolutionary Histories of Four North American Dove Species. Animals: an open access journal from MDPI, 11(9), 2677. https://doi.org/10.3390/ani11092677

Lavretsky, P., Dacosta, J. M., Hernández-Baños, B. E., Engilis, A., Sorenson, M. D., & Peters, J. L. (2015). Speciation genomics and a role for the Z chromosome in the early stages of divergence between Mexican ducks and mallards. Molecular Ecology, 24(21), 5364–5378. doi:10.1111/mec.13402
